# Effects of the FIFA 11 + Program on Physical Fitness in Youth and Adult Soccer Players: A Systematic Review and Meta-analysis

**DOI:** 10.1007/s40279-025-02346-8

**Published:** 2025-11-25

**Authors:** Ibnu Noufal Kambitta Valappil, Karuppasamy Govindasamy, Gavoutamane Vasanthi, Masilamani Elayaraja, Cain C. T. Clark, Koulla Parpa, Borko Katanic, Hüseyin Şahin Uysal, Hassane Zouhal, Urs Granacher

**Affiliations:** 1 Department of Physical Education and Sports, Pondicherry University, Puducherry, 605014 India; 2 Department of Sports, Recreation and Wellness, Symbiosis International (Deemed University), Hyderabad Campus, Modallaguda (V), Nandigama (M), Rangareddy, Telangana 509217 India; 3 College of Life Sciences, Birmingham City University, Birmingham, B15 3TN UK; 4 Faculty of Sport and Exercise Science, UCLan University of Cyprus, 7080 Pyla, Cyprus; 5 Montenergin Sports Academy, Podgorica, Montenegro; 6 Department of Physical Education and Sport, Faculty of Sport Sciences, Burdur Mehmet Akif Ersoy University, 15030 Burdur, Turkey; 7 Institut International des Sciences du Sport (2I2S), 35850, Irodouer, France; 8 Laboratoire optimisation de la performance sportive, centre national de la médecine et des sciences des sports, (LR09SEP01) Tunis, Tunisia; 9 Department of Sport and Sport Science, Exercise and Human Movement Science, University of Freiburg, 79102 Freiburg, Germany

## Abstract

**Background:**

Soccer is a high-intensity sport that requires high levels of physical fitness, including balance, change of direction (CoD), speed and power. The FIFA 11 + program has been widely promoted to enhance physical fitness and reduce injury occurrence.

**Objective:**

This meta-analysis set out to examine how the FIFA 11 + program, implemented as a warm-up versus conventional warm-up (soccer-specific and alternative warm-ups), impacts physical fitness attributes in youth and adult soccer players.

**Methods:**

After a priori defined inclusion and exclusion criteria, 17 intervention studies with 611 male and female soccer players (Tier 2–4) aged 9–29 years were eligible to be included. The FIFA 11 + program implemented in the warm-up was contrasted with conventional warm-up programs (control) on outcome measures such as dynamic balance, CoD speed, linear sprint and proxies of muscle power (vertical jump height). The influence of potential moderators (e.g., training duration, frequency, session duration, age, sex, training and performance calibre) on study outcome measures was examined using subgroup analyses with the median split method.

**Results:**

Findings demonstrated small-to-moderate improvements in favour of FIFA 11 + compared with conventional warm-ups on dynamic balance (small standardized mean differences [SMDs] = 0.37, *p* < 0.001, heterogeneity [*I*^2^] = 7), CoD speed (moderate SMDs = − 0.65, *p* = 0.005, *I*^2^ = 84), and vertical jump height (small SMDs = 0.56, *p* < 0.001, *I*^2^ = 71). Results from the sub-analyses showed that, for dynamic balance, shorter training durations (< 9 weeks) produced larger effects than longer durations (≥ 9 weeks) (SMDs = 0.62 versus SMDs = 0.17). For vertical jump height, < 9 weeks also yielded greater improvements (SMDs = 0.79 versus SMDs = 0.26). In terms of weekly training frequency, ≥ 3 sessions/week elicited larger gains in change-of-direction speed (SMDs = − 1.05 versus SMDs = − 0.12) and vertical jump height (SMDs = 0.73 versus SMDs = 0.01) compared with < 3 sessions/week. Regarding participant characteristics, players aged ≥ 18 years showed greater improvements than those < 18 years in change-of-direction speed (SMDs = − 1.45 versus SMDs = − 0.06) and vertical jump height (SMDs = 0.64 versus SMDs = 0.22). For sex differences, males experienced greater benefits than females in change-of-direction speed (SMDs = − 0.79 versus SMDs = − 0.04) and vertical jump height (SMDs = 0.54 versus SMDs = 0.09). Finally, higher-level players (≥ tier 3) demonstrated greater improvements in vertical jump height than lower-tier players (< Tier 3) (SMDs = 0.75 versus SMDs = 0.01). The observed benefits were statistically significant but generally of small-to-moderate magnitude and affected by study heterogeneity and program implementation differences. Most studies included male participants, limiting generalizability to female and underrepresented populations. Several studies also lacked rigorous methodological design, particularly in allocation, concealment and blinding. Reporting of training and demographic variables was often incomplete. These limitations highlight the necessity for rigorously designed, low-bias randomized controlled trials with standardized implementation of the FIFA 11 + program and thorough reporting to enhance the reliability of causal conclusions and improve clinical interpretation.

**Conclusion:**

The FIFA 11 + program was more effective than conventional soccer warm-up programs to enhance soccer players’ physical fitness (i.e., dynamic balance, CoD speed, vertical jump height). A shorter training duration (< 9 weeks) and more weekly training sessions (≥ 3 sessions/week) induced larger performance effects. FIFA 11 + was more effective in older (≥ 18 years) and male players and in players of higher performance calibre (≥ Tier 3). These findings underscore the versatility of the FIFA 11 + program to improve soccer players’ physical fitness, supporting its integration into the warm-up of regular soccer training sessions.

**Protocol Registration:**

https://www.crd.york.ac.uk/PROSPERO/view/CRD42025633810, PROSPERO: CRD42025633810.

**Graphical Abstract:**

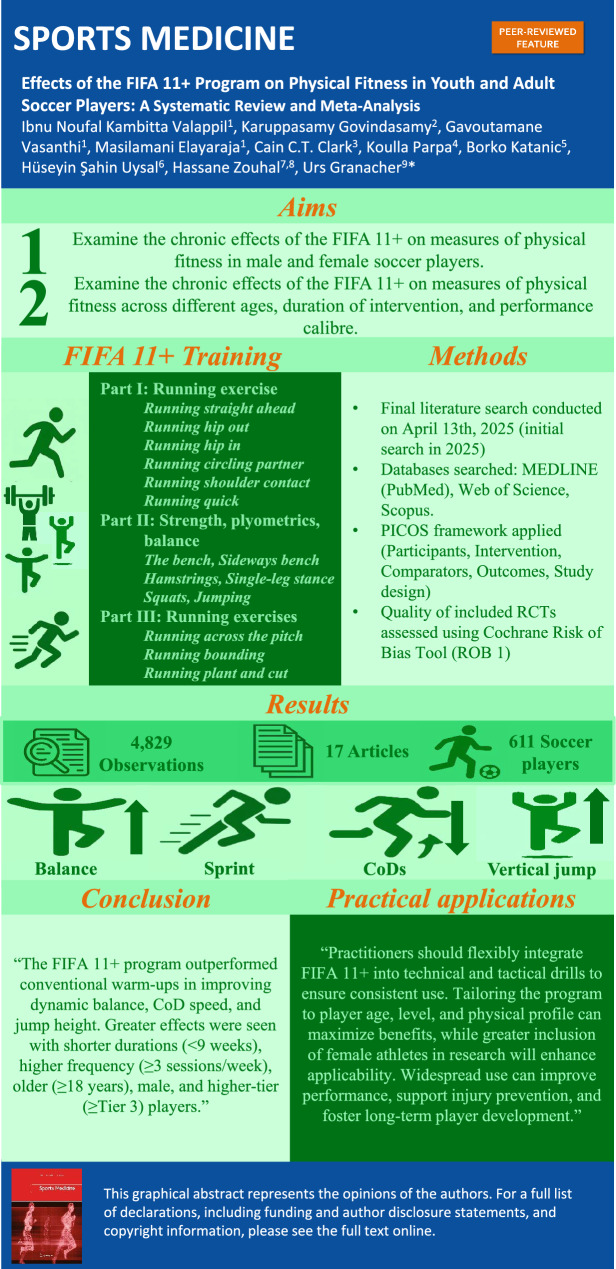

**Supplementary Information:**

The online version contains supplementary material available at 10.1007/s40279-025-02346-8.

## Key Points

**Table:** 

The FIFA 11 + program has primarily been applied to prevent injuries in youth and adult soccer. Less is known about the performance-enhancing effects of FIFA 11 + when implemented as part of a warm-up.
This meta-analysis revealed that FIFA 11 + is effective in improving dynamic balance, linear sprint, change of direction speed and vertical jump height in youth and adult male and female soccer players aged 9–29 years.
FIFA 11 + seems to be particularly effective with a training duration of < 9 weeks and ≥ 3 weekly training sessions. FIFA 11 + was more effective in older (≥ 18 years) and male players, and in players of higher performance calibre (≥ Tier 3).

## Introduction

Optimizing player preparation strategies remains a key focus in soccer, with growing interest in how structured warm-up routines may impact on performance enhancement and injury prevention [1, 2]. Evidence suggests that well-designed warm-up programs can improve neuromuscular control, enhance agility and reduce the incidence of non-contact injuries [3, 4]. Moreover, integrating sport-specific exercises within these routines may lead to acute performance benefits and long-term physiological adaptations [5–7]. In 2006, the World Soccer Association (FIFA) developed a training program called FIFA 11 + , which contains the multimodal components of neuromuscular training [8]. The FIFA 11 + program is particularly suited to be included as a warm-up program in soccer-specific training sessions in youth and adult male and female soccer players [9–12]. Originally developed as an injury prevention program, the FIFA 11 + program has also been effective in enhancing players’ physical fitness [13]. It is important to note that these performance benefits may arise through two distinct pathways: (1) acute effects, such as neuromuscular facilitation and improved readiness when the program is applied immediately as a warm-up [5, 6, 14], and (2) chronic adaptations, including long-term improvements in measures of strength, balance and power when the program is implemented consistently over several weeks [4]. Considering the well-established chronic benefits of the FIFA 11 + program for injury prevention and performance enhancement, it has been adopted by different sports such as volleyball [15], basketball [16, 17], rugby [18, 19] and futsal [3, 20], becoming a cornerstone of pre-match preparation and training. The performance-enhancing effects of the FIFA 11 + are believed to be underpinned by neuromuscular adaptations, including improved motor unit recruitment, increased lower-limb and core muscle strength, enhanced proprioception and better intermuscular coordination [21–23]. These adaptations collectively contribute to superior balance, faster and more efficient change-of-direction movements, and increased jumping and sprinting capabilities [4, 24].

The most recent systematic review and meta-analysis examining the chronic effects of the FIFA 11 + on physical fitness, particularly change of direction (CoD) speed in soccer and futsal, was conducted in 2021 [24]. The analysis included 15 studies, and the authors concluded that the FIFA 11 + training program has the potential to significantly enhance CoD performance. However, the previous review incorporated five studies involving futsal players, whereas the present study focuses exclusively on soccer players. It is important to note that futsal differs substantially from soccer in terms of physiological demands, match-play characteristics, technical-tactical requirements and overall game dynamics, which may limit the generalizability of findings across the two sports [25–27]. Given that, 4 years have passed since the last meta-analysis on this topic, a re-evaluation of the scientific literature is warranted to provide a more comprehensive and up-to-date synthesis of the available evidence on FIFA 11 + effectiveness.

While the FIFA 11 + has shown overall positive effects on physical fitness [4], the magnitude of these benefits may depend on contextual factors such as training frequency, program duration, player age, sex and performance calibre [28, 29]. For example, higher training frequency could accelerate neuromuscular adaptations [23], older players may respond differently due to greater training experience and physical maturity, and the performance level of a player may impact on the capacity to translate training stimuli into performance gains [30]. However, these moderating influences remain insufficiently explored in the existing literature. In this study, training and performance calibre was prioritized as a key moderator because players’ competitive tier reflects not only their technical-tactical proficiency but also their physical conditioning and adaptability to structured training, making it a critical determinant of how effectively the FIFA 11 + program translates into performance outcomes [14, 31]. In contrast, variables such as session duration primarily reflect total program exposure rather than athletes’ intrinsic adaptive capacity. For example, longer warm-up protocols may increase training load without yielding greater performance improvements if baseline fitness and skill level remain unaccounted [14]. Focusing on performance calibre allows the analysis to capture how athletes’ preparedness and adaptive capacity influence the effectiveness of the FIFA 11 + program. Addressing this gap through subgroup analyses can provide a deeper understanding of the conditions under which the FIFA 11 + is most effective, thereby informing more targeted implementation strategies for different player populations.

In this systematic review with meta-analysis, we aimed to examine the chronic effects of the FIFA 11 + on measures of physical fitness in male and female soccer players across different ages and performance levels, with specific fitness components identified on the basis of the outcomes reported in the included studies. Findings from this study will not only update the available knowledge base on the effectiveness of FIFA 11 + but also provide actionable insights for coaches, trainers and sports scientists seeking to enhance soccer performance through improvements in dynamic balance, agility, neuromuscular control and injury resilience.

## Methods

This systematic review was carried out in accordance with the recommendations provided by the Preferred Reporting Items for Systematic Reviews and Meta-Analyses [32], and it was filed in advance in the PROSPERO register for systematic reviews (reference: CRD42025633810).

### Literature Search Strategy

A computerized final literature search was conducted on 13 April, 2025 (Initial search conducted in 2025 and updated on 13 April, 2025) with two independent reviewers (I.N.K.V. and K.G.) systematically evaluating the literature searches. The search was performed across three major databases: MEDLINE (PubMed), Web of Science and Scopus [33]. To ensure a comprehensive review, keywords were selected on the basis of expert opinions and an extensive evaluation of the relevant literature [34]. Manual searches of the reference lists of earlier published systematic reviews were also conducted to locate other papers [35]. Boolean logic was employed to structure search queries, incorporating various keyword phrases, MeSH terms and combinations thereof. The keywords utilized for the search syntax included the following: ‘FIFA 11 + ,’ ‘FIFA 11 plus,’ ‘FIFA 11 + program*,’ ‘11 + ,’ ‘FIFA 11 plus program*,’ ‘football player*,’ ‘soccer player*,’ ‘football,’ ‘soccer,’ ‘speed,’ ‘sprint,’ ‘agility,’ ‘change of direction,’ ‘change-of-direction,’ ‘COD,’ ‘balance,’ ‘jump,’ ‘plyometric*,’ ‘motor skills,’ ‘muscle power’ and ‘athletic performance.’ These terms were carefully selected using the operators AND and OR to capture the breadth of research relevant to the study's focus. Table 1 provides a detailed summary of the three distinct database search strategies employed in the review.

**Table 1:** Detailed search strategies from selected databases

Database	Search strategy
SCOPUS	(‘FIFA 11 + ’ OR ‘FIFA 11 plus’ OR ‘FIFA 11 + program*’ OR ‘11 + ’ OR ‘FIFA 11 plus program*’) AND (‘football player*’ OR ‘soccer player*’ OR ‘football’ OR ‘soccer’) AND (‘speed’ OR ‘sprint’ OR ‘agility’ OR ‘change of direction’ OR ‘change-of-direction’ OR ‘COD’ OR ‘balance’ OR ‘jump’ OR ‘plyometric*’ OR ‘motor skill*’ OR ‘muscle power’ OR ‘athletic performance’)
PUBMED	(‘FIFA 11 + ’ OR ‘FIFA 11 plus’ OR ‘FIFA 11 + program*’ OR ‘11 + ’ OR ‘FIFA 11 plus program*’) AND (‘football player*’ OR ‘soccer player*’ OR ‘football’ OR ‘soccer’) AND (‘speed’ OR ‘sprint’ OR ‘agility’ OR ‘balance’ OR ‘jump’ OR ‘plyometric*’ OR ‘change of direction’ OR ‘change-of-direction’ OR ‘COD’ OR ‘motor skill*’ OR ‘muscle power’ OR ‘physical fitness’ OR ‘athletic performance’[MeSH])
WEB OF SCIENCE	TS = ((‘FIFA 11 + ’ OR ‘FIFA 11 plus’ OR ‘FIFA 11 + program*’ OR ‘11 + ’ OR ‘FIFA 11 plus program*’) AND (‘football player*’ OR ‘soccer player*’ OR ‘football’ OR ‘soccer’) AND (‘speed’ OR ‘sprint’ OR ‘agility’ OR ‘change of direction’ OR ‘change-of-direction’ OR ‘COD’ OR ‘balance’ OR ‘jump’ OR ‘plyometric*’ OR ‘motor skill*’ OR ‘muscle power’ OR ‘physical fitness’ OR ‘athletic performance’))

### Inclusion and Exclusion Criteria

To ensure a systematic and rigorous selection of studies, the Participants, Intervention, Comparators, Outcomes, and Study (PICOS) design framework was utilized [36]. This approach provided a structured method for evaluating study eligibility on the basis of clearly defined criteria. The inclusion and exclusion criteria adopted in this meta-analysis were designed to identify high-quality studies relevant to the objectives, while excluding research that did not meet the necessary standards. For the purpose of this review, studies that fulfilled the criteria are presented in Table 2.

**Table 2:** Selection criteria used in the meta-analysis

Category	Inclusion criteria	Exclusion criteria
Population	Male and female soccer players aged > 9 +	Athletes from different sports other than soccer
Intervention	FIFA 11 + warm-up program	FIFA 11 + program applied as full training session and not as a warm-up; other training modalities than the FIFA 11 +
Comparator	Active control group (soccer-specific and conventional warm-up)	Passive control groups
Outcomes	At least one measure of physical fitness including dynamic balance, linear sprint and CoD speed, agility, power (vertical jump height)	No physical fitness data, only injury prevention data
Study design	Randomized controlled trials (RCTs)	Cross-sectional studies, controlled studies, case studies and observational studies

### Selection Process

**Fig. 1:**
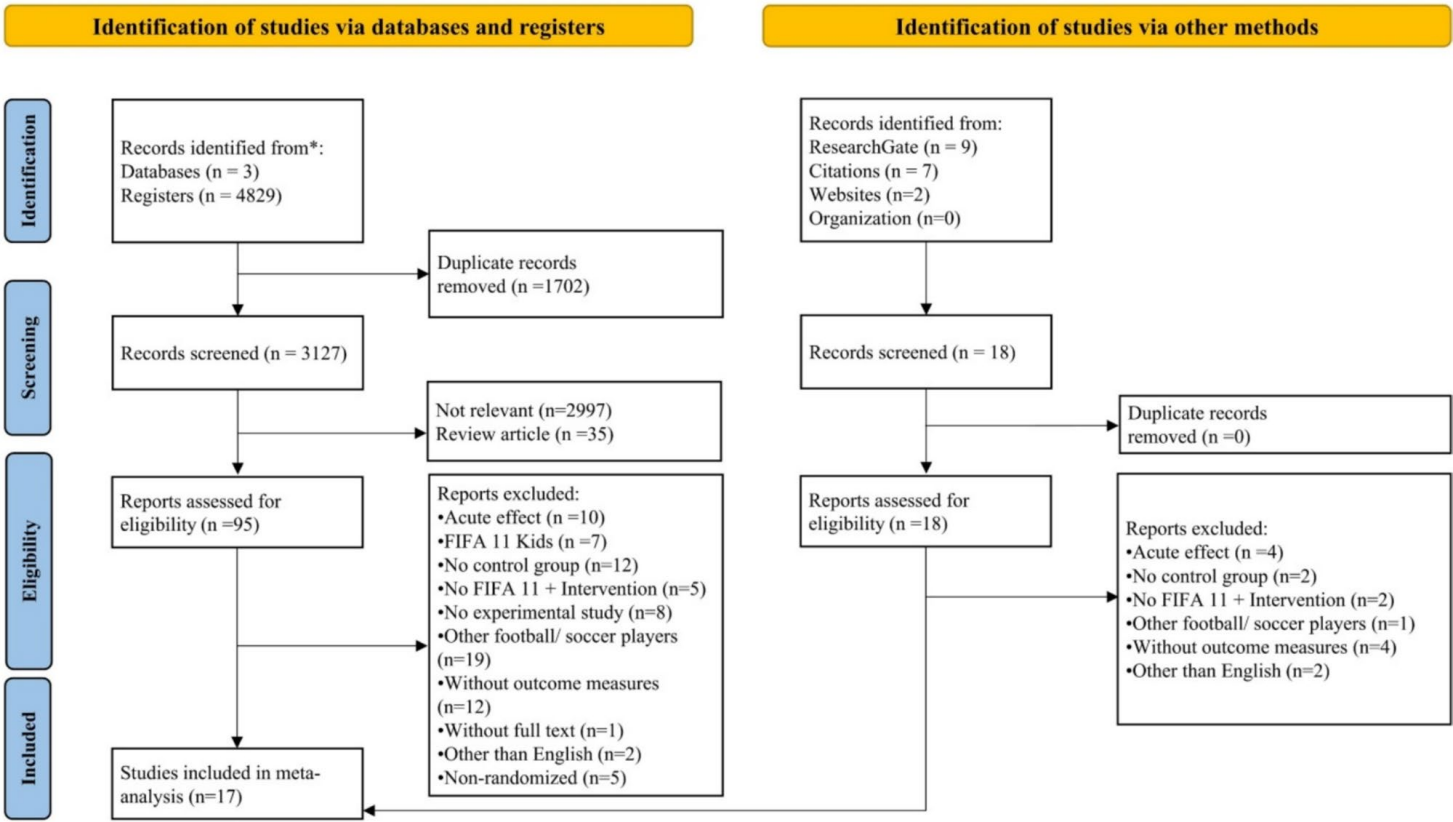
Preferred Reporting Items for Systematic Reviews and Meta-Analyses (PRISMA) flow diagram detailing identification, screening, eligibility criteria and included studies at each stage of the systematic review

Relevant data from the included studies were extracted into Microsoft Excel templates containing the titles of the articles and their corresponding DOIs. After removing duplicate records, the remaining documents were tabulated for further processing. Preliminary inclusion based on the PICOS framework (Population, Intervention, Comparison, Outcome and Study Design) was assessed collaboratively by all authors. This initial screening ensured that the studies met the predefined eligibility criteria before proceeding to detailed data extraction. Two authors (I.N.K.V. and K.G.) independently extracted key details from the included studies. These details comprised the first author’s last name, year of publication, country of origin, study design, sample size, intervention specifics (duration, frequency and follow-up), outcome measures and study findings. A third author (G.V.) verified the data for accuracy and completeness, providing an additional layer of validation [37]. Figure 1 shows the flowchart that depicts the study selection procedure for meta-analysis.

### Risk of Bias Assessment

The quality of the included randomized controlled trials (RCTs) was evaluated by two independent reviewers (I.N.K.V. and K.G.) using the Cochrane Risk of Bias Tool (ROB 1) [35]. To assess the Cochrane risk of bias, we focused on five domains: random sequence generation, allocation concealment, blinding of participants and personnel, blinding of outcome assessment, incomplete outcome data, selective reporting and other bias [38]. In addition, two authors (G.V. and M.E.) independently reviewed the risk of bias for each included study, with any disagreements resolved through discussion and consensus involving a third and last author (G.V. and U.G.). Funnel plots were additionally created to identify potential publication bias (Electronic Supplementary Material [2]).

### Data Extraction

Data pertaining to physical fitness, including measures of muscle power, linear sprint and CoD speed, agility and balance [39], were retrieved from each included research article. Dynamic balance was assessed using tests such as the Y balance test (YBT) or the star excursion balance test (SEBT). As proxies of muscle power, vertical jump height was assessed as indicated by the countermovement jump (CMJ), the drop jump (VDJ), the squat jump (SJ) test and the Bosco index. CoD speed and agility were evaluated through the T-Test, the Illinois Agility Test (IAT), the modified agility test (MAT), the arrow agility test (AAT) and the V-cut test. Linear sprint speed was assessed using the 10-m, 20-m and 30-m sprint tests. One author (I.N.K.V.) extracted the means, standard deviations (SD) and sample sizes (*n*) from the publications that were included in the study, and a second author (K.G.) verified the accuracy of the retrieved data. The results were presented in the Electronic Supplementary Material [1]. In the event that there was a disagreement between the researchers, it was settled by conversation with a fourth author (M.E.). These characteristics included the participant’s age, total duration of the intervention, training frequency, duration of the training session, participant’s age, type of training protocol and the outcome measures that were utilized in the study. These characteristics are presented in Table 3.

**Table 3:** Subgroup characteristics

Study	Duration	Frequency /week	Age	Sex	Tier level
Akbari et al. [63]	< 9 weeks	≥ 3 sessions	< 18 years	Male	4
Arede et al. [10]	< 9 weeks	< 3 sessions	< 18 years	Male	3
Asgari et al. [54]	≥ 9 weeks	≥ 3 sessions	< 18 years	Male	2
Ayala et al. [55]	< 9 weeks	≥ 3 sessions	< 18 years	Male	2
Costa Silva et al. [59]	≥ 9 weeks	≥ 3 sessions	≥ 18 years	Male	3
Daneshjoo et al. [56]	< 9 weeks	< 3 sessions	≥ 18 years	Male	2
Daneshjoo et al. [13]	< 9 weeks	≥ 3 sessions	≥ 18 years	Male	2
Foqha et al. [53]	≥ 9 weeks	≥ 3 sessions	≥ 18 years	Male	3
Hwang et al. [64]	≥ 9 weeks	≥ 3 sessions	≥ 18 years	Male	2
Impellizzeri et al. [61]	≥ 9 weeks	≥ 3 sessions	≥ 18 years	Male	3
Nawed et al. [52]	≥ 9 weeks	≥ 3 sessions	≥ 18 years	Male	3
Pardos-Mainer et al. [11]	≥ 9 weeks	< 3 sessions	< 18 years	Female	2
Parsons et al. [12]	≥ 9 weeks	< 3 sessions	< 18 years	Female	2
Robles-Palazón et al. [58]	< 9 weeks	≥ 3 sessions	< 18 years	Male	2
Sharifatpour et al. [9]	≥ 9 weeks	≥ 3 sessions	≥ 18 years	Male	3
Zarei et al. [57]	≥ 9 weeks	< 3 sessions	< 18 years	Male	2
Zhou et al. [62]	< 9 weeks	≥ 3 sessions	< 18 years	Male	2

Tier level 2 = trained/developmental, Tier level 3 = highly trained/national level, Tier level 4 = elite/international level

### Statistical Analyses

The Review Manager Software (RevMan 5.4) was used to meta-analyse our findings [40]. The threshold for statistical significance was established at *p* < 0.05. The inverse-variance random-effects model was employed, which assigns weights to each trial inversely proportional to the variance of their effect estimates, thereby accounting for both within-study and between-study variability [41]. This approach facilitates a more generalizable estimate of the overall effect by incorporating potential heterogeneity across studies. The standardized mean differences (SMDs) alongside confidence intervals (95% CI) were used to describe the magnitude of the observed effects. The SMD was interpreted using the following thresholds: < 0.2 trivial; 0.2–0.6, small; 0.6–1.2, moderate; 1.2–2.0, large; 2.0–4.0, very large; > 4.0, extremely large [42–44]. To evaluate study heterogeneity among the group and subgroup analyses, heterogeneity (*I*^2^) statistics were computed, with values categorized as follows: < 25% indicating low heterogeneity, 25–75% representing moderate heterogeneity, and > 75% reflecting high heterogeneity [45, 46]. Only outcome variables assessed in a minimum of five independent studies were included in the meta-analysis to ensure adequate statistical power, enhance the reliability of the findings and minimize the risk of drawing conclusions from limited evidence [47, 48].

Subgroup analyses were conducted to explore factors potentially moderating the effectiveness of FIFA 11 + . Programming variables were categorized using the median split method, as outlined in prior research [49], to facilitate meaningful comparison across studies, as it provides a straightforward and consistent way to classify continuous variables into balanced groups. The median was calculated if at least three studies provided data for a given moderator [50]. The following training-related factors were included: training duration (≥ 9 weeks versus < 9 weeks), weekly training frequency (≥ 3 sessions versus. < 3 sessions), training and performance calibre (≥ Tier 3 versus < Tier 3), duration of a single training session (≥ 25 min versus < 25 min). The Participant Classification Framework (based on the training and performance calibre) was applied to define tier levels consistently, whereby Tier 1 is recreationally active, Tier 2 trained/developmental, Tier 3 highly trained/national level and Tier 4 elite/international level [51]. Age was classified on the basis of developmental stages, distinguishing between adults (≥ 18 years) and youth (< 18 years), while sex was categorized as male and female. These analyses were undertaken only when at least two studies were available in each subgroup to ensure meaningful comparisons. This approach provided insights into the consistency of the findings and the potential impact of specific variables on the intervention outcomes, ensuring a robust understanding of the results.

## Results

The initial search identified 4829 articles from three databases and 18 from other sources (e.g., ResearchGate, Citations, Websites). After removing 1702 duplicates, 3127 articles remained for eligibility assessment. Title and abstract screening excluded 35 review articles and 2997 unrelated studies. This left 95 database-sourced articles and 18 from other sources for further evaluation. Full-text screening led to the exclusion of 96 articles on the basis of predefined PICOS criteria. Ultimately, 17 studies met the eligibility criteria and were included in the meta-analysis, as illustrated in Fig. 1. Notably, compared with the previous review [24], an additional nine studies that were either not included earlier or have been recently published were incorporated in the present analysis, thereby extending and updating the available evidence base.

### Study Characteristics

The study characteristics are presented in Table 4. The included studies reported training durations ranging from 4 to 30 weeks, with training frequencies varying between 1 and 5 weekly sessions. Player’s ages ranged from 9 to 29 years. A total of 611 soccer players were analysed in this meta-analysis, comprising 532 males (15 studies) and 79 females (two studies). Soccer players’ training and performance calibre ranged from Tier 2 to Tier 4, with 334 soccer players being rated as < Tier 3 (seven studies), 277 ≥ Tier 3 (ten studies). A total of 16 articles reported intervention durations ranging from 15 to 30 min per session. At the same time, one study did not specify the duration details [9]. Eight studies were classified as ≥ 18 years and nine as < 18 years. By training and performance calibre, seven studies were ≥ Tier 3 and ten < Tier 3. These subgroup analyses highlight the impact of demographic and training programming variables on FIFA 11 + effectiveness, as shown in Table 3.

**Table 4:** Study characteristics

Study	Country	*n*	Age (years)	Players (level/sex)	Duration (week)	Intervention/duration of intervention per session	Comparator	F/w	Specific physical fitness outcomes
Akbari et al. [63]	Iran	FG = 12 CG = 12	15–18	Elite soccer players	8	FIFA 11 + /25 min	Regular warm-up	3	Vertical jump height (VJ) ↑*
Arede et al. [10]	Portugal	FG = 15 CG = 15	10–14	Young soccer players	4	FIFA 11 + /20 min	Regular warm-up	3	Speed (20 m↓*) CoD speed (T-test) ↓* Balance (YBT) ↑* Vertical jump height (VJ and CMJ) ↑*
Asgari et al. [54]	Iran	FG = 29 CG = 30 MG = *31*	16–18	Youth football players	16	FIFA 11 + /25 min	Regular warm-up	3	CoD speed (IAT) ↓*
Ayala et al. [55]	Spain	FG = 10 CG = 11	16–18	Youth football players	4	FIFA 11 + /25 min	Regular warm-up	3	Speed (10↑ and 20 m↓*) CoD speed (IAT) ↓* Balance (YBT) ↑* Vertical jump height (VDJ) ↑*
Costa Silva et al. [59]	Brazil	FG = 10 CG = 10	16–20	Junior soccer players	9	FIFA 11 + /20 min	Normal warm-up	3	Vertical jump height (CMJ and SJ) ↑*
Daneshjoo et al. [56]	Malaysia	FG = 12 CG = 12 HG = *12*	18–22	Soccer players	8	FIFA 11 + /20 min	Regular warm-up	3	Balance (SEBT) ↑*
Daneshjoo et al. [13]	Malaysia	FG = 12 CG = 12 HG = *12*	17–20	Young soccer players	8	FIFA 11 + /20 min	Regular warm-up	3	Speed (10 m and 20 m) ↓* CoD speed (IAT) ↓* Vertical jump height (VJ) ↑*
Foqha et al. [53]	Tunisia	FG = 13 CG = 12	24–29	Seven-a-side Soccer Athletes	10	FIFA 11 + /20 min	Regular warm-up	3	Speed (10 ↓*and 20 m↑) CoD speed (MAT) ↓*
Hwang et al. [64]	South Korea	FG = 10 CG = 10	19–21	Collegiate soccer players	12	FIFA 11 + /25 min	Normal warm-up	5	CoD speed (AAT-R and L) ↓* Speed (10 and 30-m ↓*)
Impellizzeri et al. [61]	Italy	FG = 42 CG = 39	19–28	Football players	9	FIFA 11 + /25 min	Regular warm-up	3	Speed (20 m) ↓ CoD speed (T-Test) ↓ Balance (SEBT) ↑ Vertical jump height (VJ) ↑
Nawed et al. [52]	India	FG = 29 CG = 28	18–23	Soccer players	12	FIFA 11 + /25 min	Regular warm-up	5	Speed (20 m) ↓* CoD speed (T-test, and IAT) ↓ Vertical jump height (VJ) ↑*
Pardos-Mainer et al. [11]	Spain	FG = 19 CG = 17	12–14	Adolescent soccer players	10	FIFA 11 + /15 min	Standard warm-up	2	CoD speed (V-cut Test) ↑* Balance (YBT) ↑* Vertical jump height (CMJ) ↑*
Parsons et al. [12]	Canada	FG = 25 CG = 18	9–11	Youth soccer players	20 (5-month)	FIFA 11 + /30 min	Regular warm-up (Coach determined)	1–2	Balance (YBT) ↓ CoD speed (T-Test) ↓ Jump High (VJ)↑
Robles-Palazón et al. [58]	Spain	FG = 10 CG = 11	15–18	Youth football players	4	FIFA 11 + /25 min	Regular warm-up	3	Balance (YBT) ↑ Speed (10-m↑and 20-m↓) Jump High (VJ) =
Sharifatpour et al. [9]	Iran	FG = 20 CG = 20	22–28	Beach soccer player	12	FIFA 11 + /–	General warm-up	3	Jump High (Bosco index) ↑*
Zarei et al. [57]	Iran	FG = 34 CG = 32	14–16	Adolescent soccer players	30	FIFA 11 + /25 min	Standard warm-up	2	CoD speed (IAT) ↓* Speed (9.1-m and 36.6 m↓*) Jump High (VJ) ↑*
Zhou et al. [62]	China	FG = 10 CG = 10	10–15	Youth soccer players	8	FIFA 11 + /20 min	Regular warm-up	3	Speed (30 m) ↓* CoD speed (SR) ↓*

*YBT* Y balance test, *VJ* vertical jump, *MAT* modified agility test, *IAT* Illinois agility test, *SR* Shuttle run test, *SEBT* star excursion balance test, *VJ* vertical jump, *CMJ* countermovement jump, *AAT* arrow agility test, *SJ* squat jump, *CoD* change of direction, ↑ Value increased, ↑* Value significantly increased, ↓ Value decreased, ↓* Value significantly decreased, *FG* FIFA 11 + group, *CG* control group, *MG* modified FIFA 11 + group, *HG* HarmoKnee group, – not mentioned, *F/w* frequency per week, *n* number of participant in each group

### Methodological Quality of the Included Studies

**Fig. 2:**
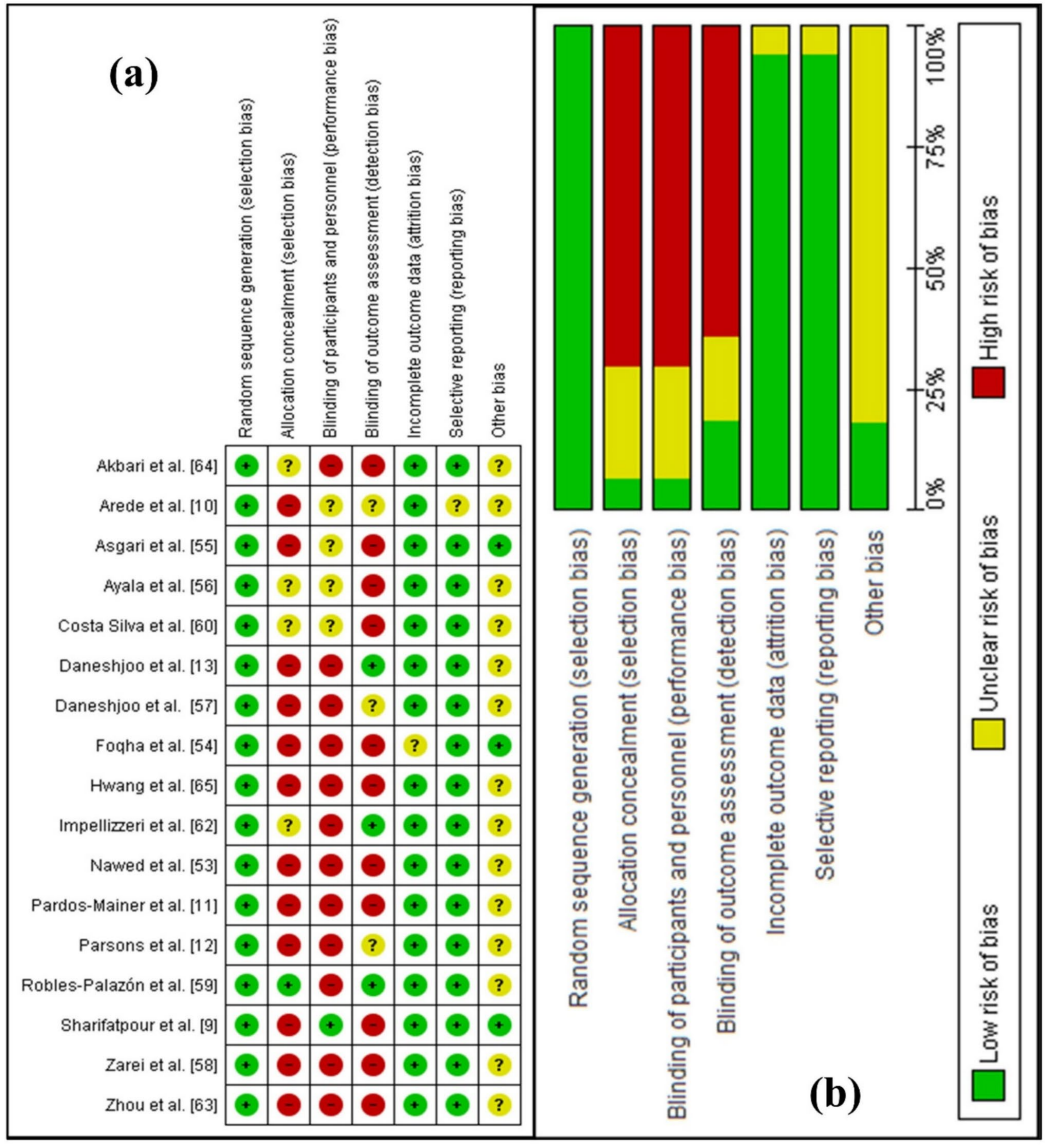
Representation of Cochrane Risk of Bias Tool-1 (ROB1) summary: **a** ROB1 for individual studies, **b** Overall ROB1

The studies demonstrate varying levels of bias across different domains (Fig. 2). Random sequence generation was assessed as having a low risk of bias in all the included studies [9–13, 52–64]. Allocation concealment was frequently rated as unclear or high risk, reflecting insufficient reporting or inadequate methods. The blinding of participants and personnel presented the highest proportion of high-risk ratings, highlighting significant shortcomings in implementing blinding protocols. In contrast, blinding of outcome assessment generally showed a high risk of bias [9, 10, 12, 13, 52, 54, 55, 57, 59, 62–64], except for a few studies [56, 58, 61]. A total of 17 studies were included in the meta-analysis, with the overall risk of bias assessed using the ROB 1 tool, as illustrated in Fig. 2.

### Effects of FIFA 11 + on Measures of Balance

**Fig. 3:**
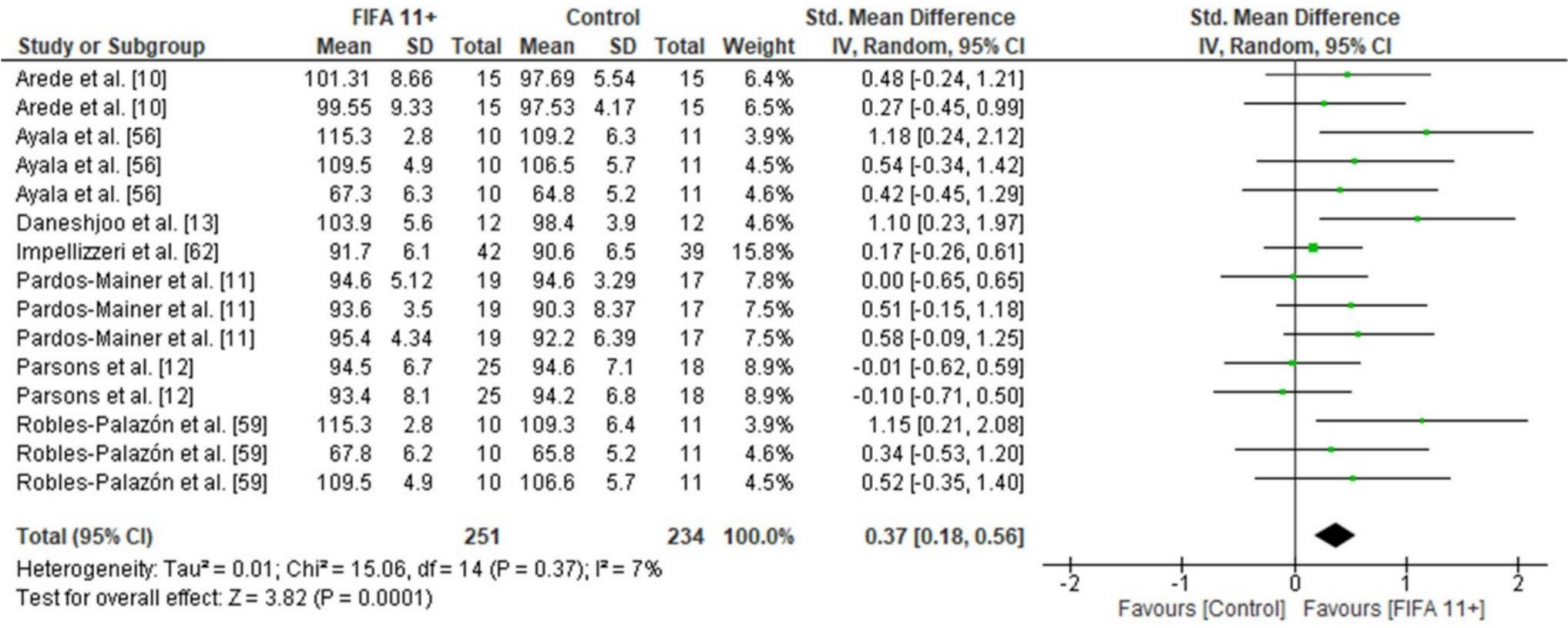
Forest plot on the effects of FIFA 11 + versus active controls on measures of balance in soccer players

A statistically significant difference was observed in favour of the FIFA 11 + for dynamic balance (seven studies: small SMDs = 0.37, CI 0.18–0.56, *p* < 0.001, *I*^2^ = 7) (Fig. 3).

### Effects of FIFA 11 + on Linear Sprint and Change-of-Direction Speed

**Fig. 4:**
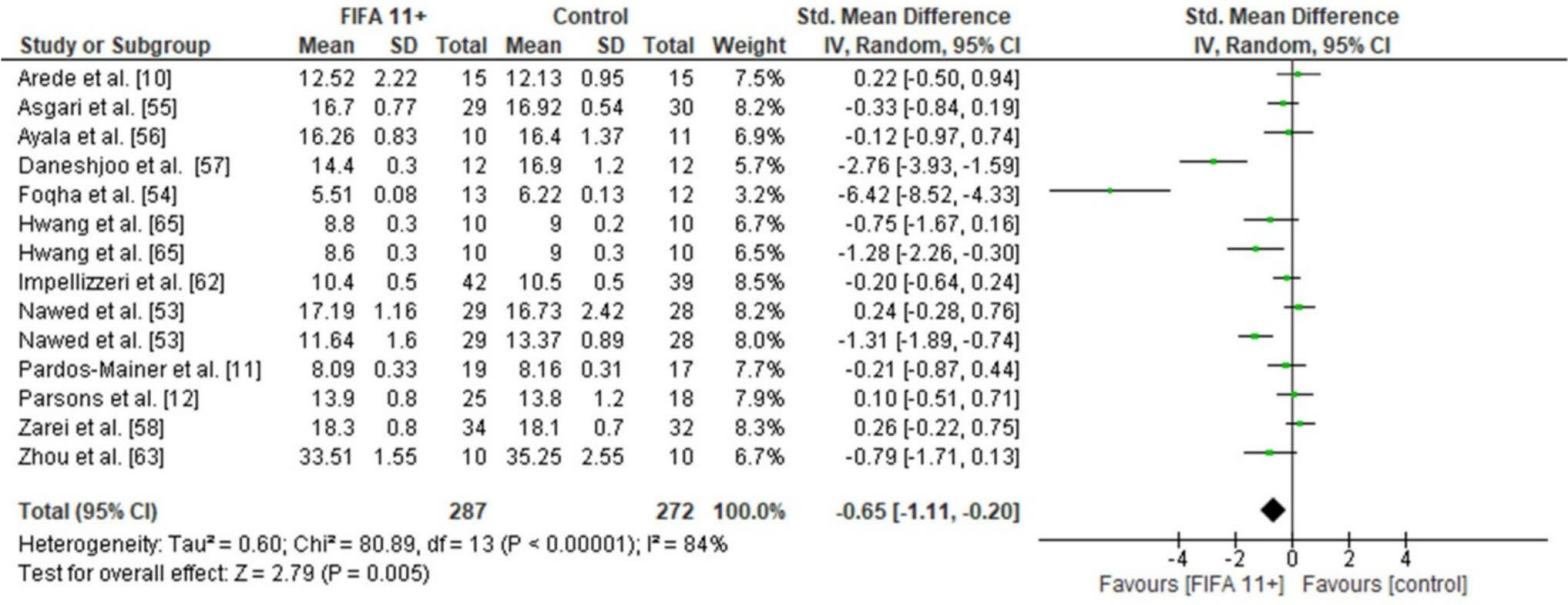
Forest plot on the effects of FIFA 11 + versus active controls on measures of change-of-direction speed in soccer

**Fig. 5:**
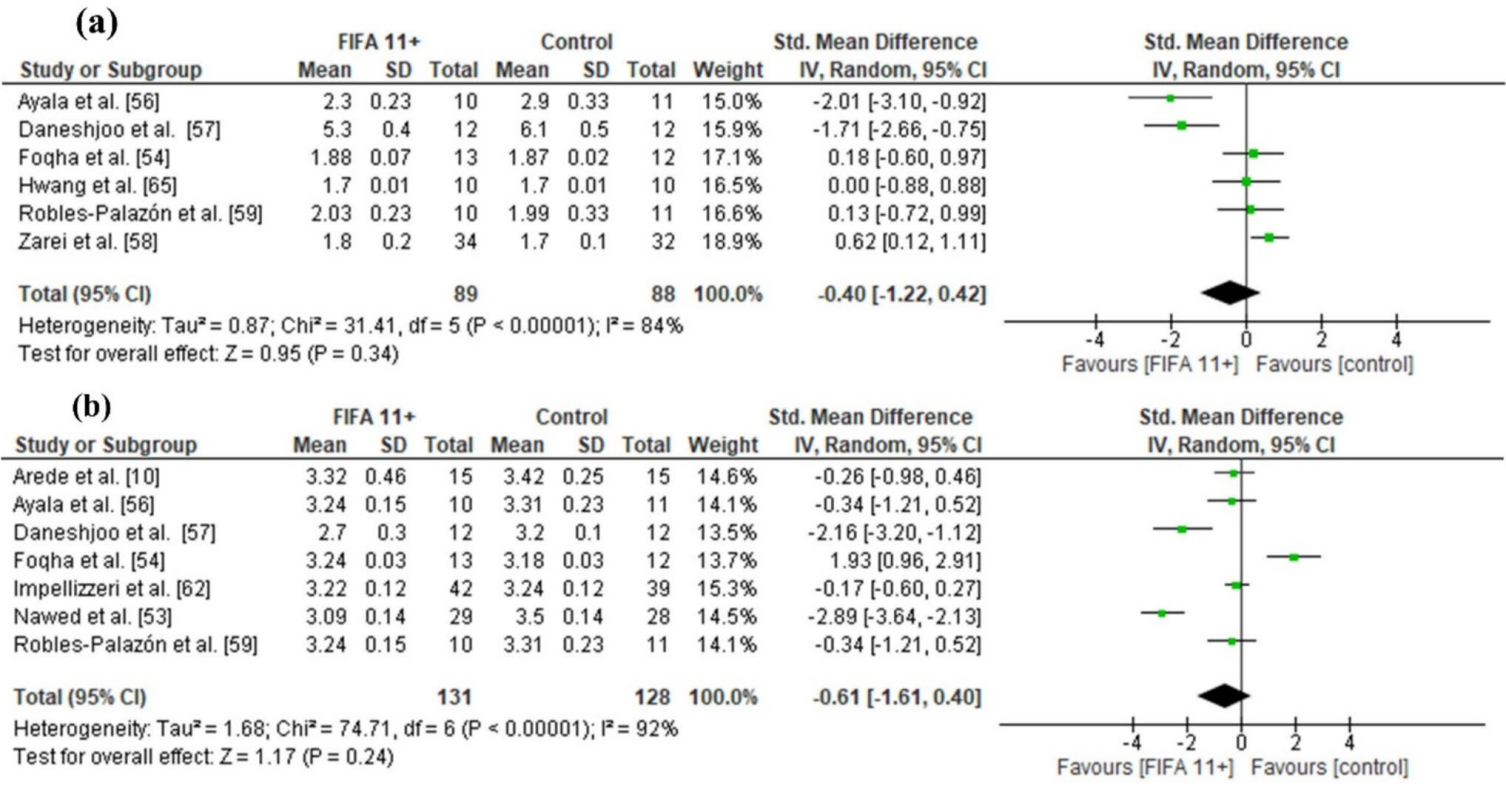
Forest plot on the effects of FIFA 11 + versus active controls on measures of linear sprint **a** 10-m and **b** 20-m in soccer players

FIFA 11 + induced significantly larger CoD speed improvements than active controls were more effective (12 studies: moderate SMDs = − 0.65, CI − 1.11, − 0.20, *p* = 0.005, *I*^2^ = 84) (Fig. 4). No statistically significant difference was observed between the FIFA 11 + group and the control group for 10-m linear sprint speed (six studies: small SMDs = − 0.40, CI − 1.22 to 0.42, *p* = 0.34, *I*^2^ = 84) (Fig. 5a) and 20-m linear sprint speed (seven studies: moderate SMDs = 0.61, CI 1.61 to 0.40, *p* = 0.24, *I*^2^ = 92) (Fig. 5b).

### Effects of FIFA 11 + on Proxies of Muscle Power

**Fig. 6:**
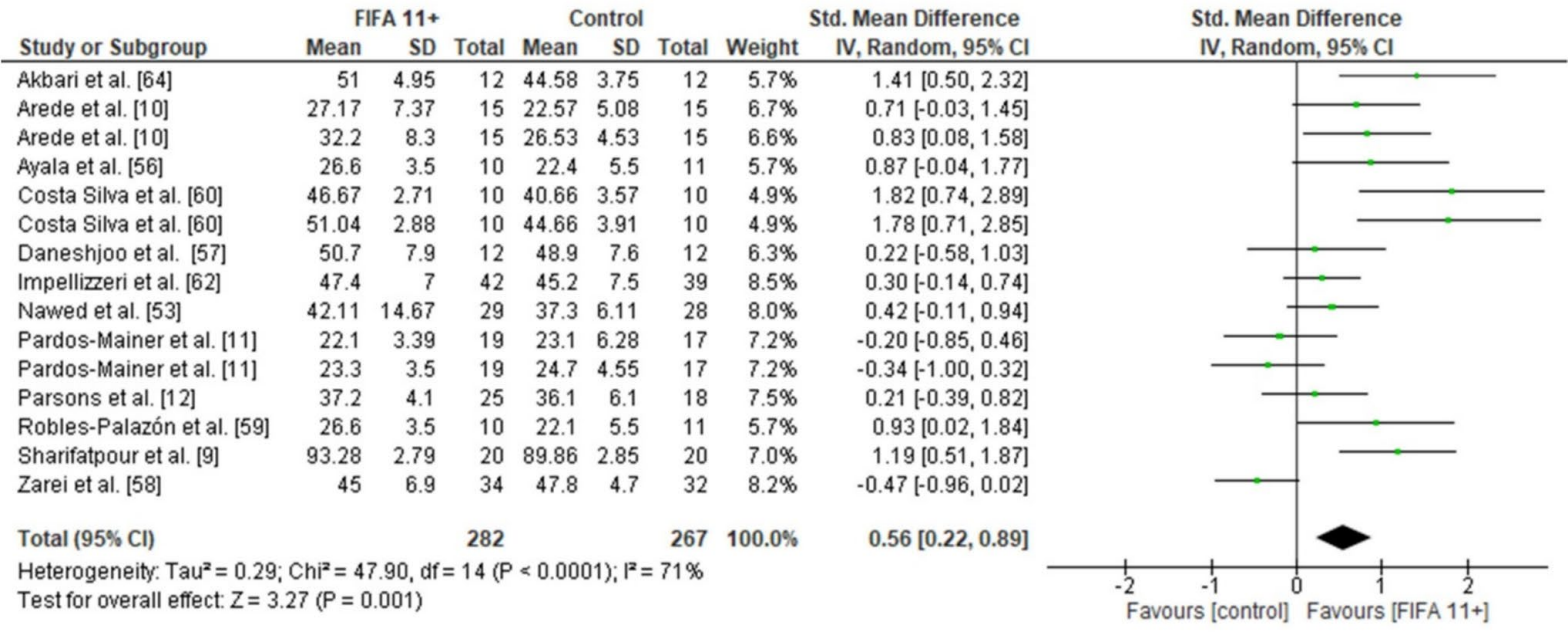
Forest plot on the effects of FIFA 11 + versus active controls on measures of muscle power (vertical jump height) in soccer

Vertical jump height was assessed as a proxy of muscle power. A statistically significant difference was observed between the FIFA 11 + group and the active control group for vertical jump height (12 studies: small SMDs = 0.56, CI 0.22–0.89, *p* < 0.001, *I*^2^ = 71) (Fig. 6).

The overall analysis on outcome measures demonstrated low-to-high study heterogeneity, with *I*^2^ values ranging between 7 and 92%. Specifically, dynamic balance showed low heterogeneity (*I*^2^ = 7%), vertical jump height reflected moderate heterogeneity (*I*^2^ = 71%), while change-of-direction speed (*I*^2^ = 84%) and linear sprint outcomes indicated high heterogeneity (10-m sprint: *I*^2^ = 84%; 20-m sprint: *I*^2^ = 92%). These findings suggest variability in the treatment effects across the included studies, as illustrated in Figs. 3, 4, 5 and 6.

### Subgroup Analyses According to Programming and Demographic Parameters

For training duration (≥ 9 versus < 9 weeks), larger FIFA 11 + effects were found for < 9 weeks. With regards to weekly training frequency (≥ 3 versus < 3session), larger FIFA 11 + effects were found for ≥ 3 sessions. With regards to chronological age (≥ 18 versus < 18 years), larger FIFA 11 + effects were found for ≥ 18 years (i.e., adult soccer players). In terms of sex (males versus females), larger FIFA 11 + effects were found for male soccer players. For training and performance calibre (≥ Tier 3 versus < Tier 3), a larger effect was found for ≥ Tier 3 soccer players (Table 5).

**Table 5:** Subgroup analyses of potential moderators on balance, speed (10- and 20-m linear sprint and change of direction speed), muscle power (vertical jump height)

	Group (*n*)	# of studies	SMDs (95% CI)	*I*^*2*^	*p* value	*p*_diff_
*Balance outcomes*						
*Duration*						
≥ 9 weeks	6	3	0.17 (− 0.07, 0.41)	0	0.15	**0.02**
< 9 weeks	9	4	0.62 (0.34, 0.90)	0	< 0.001	
*Training frequency*						
≥ 3 sessions	7	3	0.47 (0.18, 0.75)	1	0.001	0.38
< 3 sessions	8	4	0.30 (0.04, 0.55)	14	0.02	
*Session duration*						
≥ 25 min	9	4	0.34 (0.07, 0.62)	21	0.01	0.61
< 25 min	6	3	0.45 (0.16, 0.74)	0	0.002	
*Age*						
≥ 18 years	2	2	0.56 (− 0.34, 1.45)	71	0.22	0.09
< 18 years	13	5	0.36 (0.16, 0.57)	0	< 0.001	
*Sex*						
Male	10	5	0.49 (0.26, 0.73)	0	< 0.001	0.41
Female	5	2	0.17 (− 0.11, 0.46)	0	0.23	
*Training and performance calibre*						
≥ Tier 3	3	2	0.26 (− 0.07, 0.59)	0	0.13	0.41
< Tier 3	12	5	0.43 (0.18, 0.68)	21	< 0.001	
*Change of direction speed outcomes*						
*Duration*						
≥ 9 weeks	10	8	− 0.61 (− 1.13, − 0.08)	85	0.02	0.77
< 9 weeks	4	4	− 0.80 (− 1.93, 0.34)	84	0.17	
*Training frequency*						
≥ 3 sessions	10	8	− 1.05 (− 1.68, − 0.42)	86	0.001	**0.001**
< 3 sessions	4	4	0.12 (− 0.18, 0.42)	0	0.44	
*Session duration*						
≥ 25 min	9	7	− 0.32 (− 0.70, 0.06)	71	0.10	0.07
< 25 min	5	5	− 1.72 (− 3.21, − 0.24)	92	0.02	
*Age*						
≥ 18 years	7	5	− 1.45 (− 2.37, − 0.53)	91	0.002	**0.004**
< 18 years	7	7	− 0.06 (− 0.30, 0.18)	4	0.63	
*Sex*						
Males	12	10	− 0.79 (− 1.33, − 0.25)	86	0.004	**0.04**
Females	2	2	− 0.04 (− 0.49, 0.40)	0	0.84	
*Training and performance calibre*						
≥ Tier 3	5	4	− 1.00 (− 2.04, 0.03)	92	0.06	0.41
< Tier 3	9	8	− 0.53 (− 1.00, − 0.06)	73	0.03	
*10-m linear sprint speed outcomes*						
*Duration*						
≥ 9 weeks	3	3	0.40 (0.03, 0.78)	0	0.04	**0.03**
< 9 weeks	3	3	− 1.17 (− 2.53, 0.20)	83	0.10	
*Session duration*						
≥ 25 min	4	4	− 0.24 (− 1.23, 0.75	84	0.64	0.64
< 25 min	2	2	− 0.74 (− 2.59, 1.11)	89	0.42	
*Age*						
≥ 18 years	3	3	− 0.48 (− 1.61, 0.65)	80	0.40	0.88
< 18 years	3	3	− 0.35 (− 1.74, 1.04)	89	0.62	
*20-m linear sprint speed*						
*Duration*						
≥ 9 weeks	3	3	− 0.39 (− 2.68, 1.90)	97	0.74	0.79
< 9 weeks	4	4	− 0.72 (− 1.51, 0.07)	70	0.07	
*Session duration*						
≥ 25 min	4	4	− 0.93 (− 2.20, 0.34)	92	0.15	0.53
< 25 min	3	3	− 0.16 (− 2.24, 1.93)	94	0.88	
*Age*						
≥ 18 years	4	4	− 0.82 (− 2.68, 1.04)	96	0.39	0.60
< 18 years	3	3	− 0.31 (− 0.77, 0.16)	0	0.19	
*Training and performance calibre*						
≥ Tier 3	4	4	− 0.36 (− 1.96, 1.23)	95	0.65	0.58
< Tier 3	3	3	− 0.91 (− 2.02, 0.20)	77	0.11	
*Vertical jump height outcome*						
*Duration*						
≥ 9 weeks	9	7	0.26 (0.05, 0.46)	78	0.01	**0.007**
< 9 weeks	6	5	0.79 (0.46, 1.13)	0	< 0.001	
*Training frequency*						
≥ 3 sessions	9	8	0.73 (0.49, 0.96)	56	< 0.001	** < 0.001**
< 3 sessions	6	4	0.01 (− 0.25, 0.27)	63	0.94	
*Session duration*						
≥ 25 min	7	7	0.29 (0.06, 0.52)	68	0.01	0.45
< 25 min	7	4	0.43 (0.14, 0.73)	74	0.004	
*Age*						
≥ 18 years	6	5	0.64 (0.37, 0.90)	68	< 0.001	**0.02**
< 18 years	9	7	0.22 (− 0.02, 0.45)	70	0.07	
*Sex*						
Male	12	10	0.54 (0.34, 0.74)	71	< 0.001	**0.003**
Female	3	2	− 0.09 (− 0.46, 0.28)	0	0.63	
*Training and performance calibre*						
≥ Tier 3	8	6	0.75 (0.52, 0.99)	58	< 0.001	** < 0.001**
< Tier 3	7	6	− 0.01 (− 0.26, 0.25)	54	0.97	

*SMD* standardized mean difference, *I*^*2*^ study heterogeneity, *p*_*diff*_ significant differences among two subgroups

Bold values indicate statistically significant subgroup differences (*p*_*diff*_ < 0.05)

## Discussion

In this meta-analysis, we aimed to synthesize evidence from 17 studies on the effects of the FIFA 11 + program on balance, and CoD speed, muscle power (jump performance) in youth and adult soccer players. Notably, authors from all included studies reported at least one positive or beneficial effect associated with the FIFA 11 + program. The results of the meta-analysis revealed that the FIFA 11 + program produced small-to-moderate improvements in dynamic balance, linear sprint and CoD speed and jump performance when compared with conventional warm-up programs (general, standard or normal warm-ups). More specifically, for measures of dynamic balance, statistically significant but small effect sizes (*p* < 0.001) were found in favour of FIFA 11 + . CoD speed improved significantly (*p* < 0.01) with moderate effect sizes, while small effects were found for vertical jump height improvements (*p* < 0.001), all in favour of FIFA 11 + . In contrast, linear sprint speed showed no statistically significant improvements following the application of FIFA 11 + .

The observed improvements in dynamic balance following the FIFA 11 + intervention may be attributed to the specific exercises included in part two of the program. This section incorporates progressive plyometric, balance and core muscle exercises, all of which are designed to enhance neuromuscular control and postural stability [65]. These exercises likely contribute to improved proprioception, postural stability and coordination, which are essential components of dynamic balance. Thus, the positive outcomes observed in this meta-analysis may be closely related to the neuromuscular adaptations elicited by these targeted training elements during the intervention [52]. Few systematic reviews have reported a positive effect on dynamic balance following various warming-up programs [7, 66, 67]. Within the sport of soccer, the ability to change directions is among the most crucial components [68]. Change of direction (CoD) ability is a multifaceted skill that relies on speed, coordination, dynamic balance, strength and reactive agility [69]. The improvements in CoD speed observed in this study may be attributed to the specific exercises in parts two and three of the FIFA 11 + program; part one includes progressive plyometric, balance and strength-based drills designed to enhance neuromuscular coordination and control. Part 2 includes different running exercises (cross-pitch running, bounding and running, plant and cut running) [65]. These exercises target essential movement qualities, such as rapid deceleration, re-acceleration and stabilization, which are critical for CoD performance [17, 70]. There has been scant evidence from systematic reviews that different warming-up programs improve CoD [7]. Although these findings support the efficacy of FIFA 11 + for improving CoD speed and balance, caution is warranted in generalizing these results to all soccer-playing populations. Most included studies featured male athletes from specific age ranges and competitive/Tier levels [9, 52, 54, 56, 57, 63, 64], and there is limited evidence on female players [11, 12], youth athletes [10, 58, 60, 62] or professional players [13, 53, 63]. Consequently, the observed performance benefits may not directly translate across all demographic and competitive groups, underscoring the need for broader, more representative research. The observed improvements in vertical jump performance following the FIFA 11 + intervention may be attributed to the progressive plyometric and strength exercises included in part two of the program. These exercises, such as planks, side planks, Nordic hamstring drills, single-leg balance activities, squats and various jumping drills (vertical, lateral and box jumps), are progressively structured to enhance core stability, lower-limb strength and explosive power [17]. By targeting neuromuscular coordination, reactive ability and biomechanical efficiency, these exercises play a crucial role in optimizing jump performance. Thus, the positive outcomes in vertical jump height observed in this study are likely a result of the neuromuscular adaptations induced by these training elements during the intervention. A small number of systematic evaluations have documented improvements in jump performance after different warm-up programs [71, 72]. However, it is important to note that such outcomes may also depend on intervention fidelity. Variability in adherence, supervision and delivery of the FIFA 11 + across studies likely influenced the magnitude of improvements observed. While some investigations ensured structured and consistent implementation, others applied it less rigorously, which may have contributed to heterogeneity in effects. These differences in fidelity and compliance should therefore be considered when interpreting pooled results [73, 74].

Subgroup analyses revealed several significant findings regarding the effects of the FIFA 11 + intervention on balance, change of direction, speed performance and jump performance among soccer players. In terms of training duration, shorter programs (< 9 weeks) significantly enhanced CoD speed and jump performance versus longer programs (≥ 9 weeks), possibly owing to their initial responsiveness to new training stimuli, whereas longer durations may lead to diminishing returns as players adapt to the regimen [75]. Training frequency played a crucial role in determining outcomes, with players training ≥ 3 sessions per week exhibiting superior improvements in CoD and vertical jump height, likely owing to consistent exposure to the intervention [76]. Interestingly, players training < 3 sessions per week achieved better results in 10 m speed, potentially because reduced frequency allowed for better recovery and adaptation specific to this metric [77]. Soccer players aged ≥ 18 years demonstrated greater improvements in CoD speed and jump performance versus those aged < 18 years, likely owing to the more advanced neuromuscular and physiological maturity of the older group, which enabled better adaptation to the training program [21, 78]. This suggests that maturity of the neuromuscular system, characterized by more efficient motor unit recruitment, enhanced coordination and improved muscle–tendon stiffness in older players [78], may provide a biological advantage in responding to structured interventions such as FIFA 11 + .

The subgroup analyses demonstrated that male versus female soccer players exhibited significantly greater improvements in CoD speed and vertical jump height, suggesting a higher neuromuscular response to the FIFA 11 + program. This may be attributed to sex-related physiological differences, such as greater muscle mass and power output in males [79]. However, sex-specific sports participation patterns may also play a role, as prior evidence indicates that female athletes often face contextual barriers (e.g., lower training adherence, fewer structured exposure hours) that could attenuate potential benefits of the training program, despite its injury-preventive and performance-enhancing capacity [80, 81]. In addition, higher training and performance calibre were associated with greater vertical jump height, with ≥ Tier 3 players showing significantly larger gains compare with < Tier 3. Notably, only one study fell under Tier 4 [63], while seven studies were classified as Tier 3. Higher-calibre players likely undergo more intensive and sport-specific training, leading to superior neuromuscular efficiency, enhanced motor unit recruitment and increased rate of force development, all of which contribute to improved maximal power production [82]. These findings may also reflect higher baseline fitness levels in elite players, which facilitate greater responsiveness to structured warm-up and plyometric components embedded within FIFA 11 + , as they can more effectively translate training stimuli into performance gains [61, 63]. Vertical jump height, reflecting athletes’ higher baseline fitness, advanced neuromuscular efficiency and greater ability to capitalize on structured plyometric training, included interventions such as the FIFA 11 + program [24, 83]. These results underscore the importance of considering factors such as training duration, frequency, age and competitive level when designing and implementing FIFA 11 + programs to improve players’ physical fitness. In addition, our findings highlight that the effects of the FIFA 11 + program may not only vary across moderators such as age, training and performance calibre but also depend on whether adaptations are acute (short-term neuromuscular responses) or chronic (long-term physiological changes). This distinction may help explain the heterogeneity observed across studies and reinforces the program’s versatility for both acute performance potentiation and sustained athletic development. Moreover, our findings suggest that the program’s effects may differ across player groups. Tailored adaptations for female athletes, considering sex-specific neuromuscular and physiological characteristics, and for youth athletes, aligned with developmental stages, could further enhance its effectiveness and ensure safe application.

### Limitations and Future Recommendations

Several limitations and recommendations for future research agendas must be acknowledged. First of all, there is a significant lack of studies on the use of the FIFA 11 + program among female soccer players. Accordingly, the current findings, mostly from male soccer players, cannot be generalized to female populations, emphasizing the necessity for research into the program’s impact on female athletes to close this gap in the literature. In addition, underrepresented populations, including youth and amateur players, should be included to improve the generalizability of findings. Furthermore, future research should strive to report more information on programming variables such as training frequency, intensity and volume, but also demographic variables including sex, chronological and biological age. By providing detailed information on these relevant issues, FIFA 11 + programs can be better targeted to the needs of the respective population under investigation. Moreover, high-quality, low-bias RCTs with standardized implementation of FIFA 11 + are needed to strengthen causal inferences. Reporting standardized outcome measures and effect sizes alongside SMDs would help clarify the clinical significance of performance improvements. Finally, many included studies lacked robust methodological rigour, particularly in terms of allocation, concealment and blinding processes. These shortcomings may cause bias that could affect the robustness of findings. Future studies should prioritize rigorous experimental designs with proper allocation concealment and blinding to enhance the credibility and generalizability of the results. In addition, heavy reliance on SMDs and high heterogeneity in certain outcomes, such as sprint performance, may limit confidence in some findings. Future research with larger sample sizes should conduct sensitivity analyses and subgroup assessments to strengthen the stability of results. In addition, although high heterogeneity was observed in the sprint outcomes (10-m and 20-m), no sensitivity analysis was conducted owing to the absence of statistically significant effects and the limited number of studies available for these measures, which could have compromised the stability of further subgroup analyses. This should be addressed in future research with larger datasets to enable more robust heterogeneity assessments.

### Practical Implications

This meta-analysis highlights the practical value of incorporating the FIFA 11 + program into routine soccer warm-ups to enhance key physical fitness attributes in both youth and adult players. Coaches, strength and conditioning professionals and physical trainers are encouraged to replace or supplement conventional warm-ups with the FIFA 11 + to improve dynamic balance, change-of-direction speed and vertical jump performance. While optimal benefits were observed when the program was performed at least three times per week and over shorter intervention periods (< 9 weeks), achieving such adherence in real-world settings may be challenging owing to factors such as match schedules, travel demands and overall training load. Therefore, practitioners should consider flexible implementation strategies, such as integrating selected FIFA 11 + exercises into technical or tactical drills, to maintain regularity. Given the substantial heterogeneity across studies, the program’s effectiveness may vary depending on age, competitive level and baseline physical characteristics, with greater effects observed in older, male and higher-tier players. However, the under-representation of female participants limits the generalizability of these findings to women’s soccer, highlighting the need for further research in more diverse and representative populations. When implemented with a context-specific approach, the FIFA 11 + can serve as an effective tool for both performance enhancement and injury prevention, supporting long-term athlete development and competitive success.

## Conclusions

This meta-analysis concluded from 17 studies, significant chronic effects of the FIFA 11 + program versus conventional soccer-specific warm-up programs on key physical fitness components, including dynamic balance, and CoD speed, as well as vertical jump performance. The observed benefits, while statistically significant, were generally of small-to-moderate magnitude and may have been influenced by variability across studies. The findings highlight the potential of FIFA 11 + to induce neuromuscular adaptations in youth and adult soccer players, resulting in improved linear sprint and CoD speed as well as muscle strength and power, important fitness qualities related to success in soccer. This study contributes to the broader field of sports science by providing robust evidence of the program’s capacity to optimize physical performance, offering valuable insights for athletes, coaches and sports organizations globally. However, the magnitude of improvement should be interpreted in light of the heterogeneity and differences in program implementation in the study. By establishing a foundation of scientifically validated benefits, the research encourages the integration of structured, evidence-based training protocols into routine practice. The demonstrated effectiveness of the FIFA 11 + program supports its adoption across diverse sports disciplines, fostering enhanced performance levels and setting new benchmarks for athletic excellence. This global perspective underscores the program’s transformative potential to redefine training strategies and contribute to the sustained growth and development of athletic performance worldwide.

## Supplementary Information

Below is the link to the electronic supplementary material.

Supplementary file1 (DOCX 53 kb)

Supplementary file2 (DOCX 206 kb)

## Declarations

### Availability of data and material

The data are provided in Table 4 and Electronic Supplementary Material [1], with further datasets available from the corresponding author upon reasonable request.
