# Supplementary material for: Effects of the FIFA 11 + Program on Physical Fitness in Youth and Adult Soccer Players: A Systematic Review and Meta-analysis
Source: Sports Med. 2025 Nov 25;56(2):521–41. doi: 10.1007/s40279-025-02346-8 (PMC12982247; doi:10.1007/s40279-025-02346-8)
Supplement: Supplementary file 1 — Supplementary file1 (DOCX 53 kb) [file 40279_2025_2346_MOESM1_ESM.docx]

**āSupplementary Material**

**Effects of the FIFA 11+ Program on Physical Fitness in Youth and Adult Soccer Players: A Systematic Review and Meta-Analysis**

**Short title:** FIFA 11+ and Physical Fitness in Soccer Players: A Meta-Analysis

Ibnu Noufal Kambitta Valappil^1^; Karuppasamy Govindasamy^2^; Gavoutamane Vasanthi^1^; Masilamani Elayaraja^1^; Cain C.T.Clark^3^; Koulla Parpa^4^; Borko Katanic^5^; Hüseyin Şahin Uysal^6^; [Hassane Zouhal](https://link.springer.com/article/10.1186/s40798-025-00831-y#auth-Hassane-Zouhal-Aff8-Aff9)^7,8^; Urs Granacher^9^**^*^**

1. Department of Physical Education and Sports, Pondicherry University, 605014 Puducherry, India
2. Department of Sports, Recreation and Wellness, Symbiosis International (Deemed University), Hyderabad Campus, Modallaguda (V), Nandigama (M), Rangareddy, 509217 Telangana, India
3. College of Life Sciences, Birmingham City University, B15 3TN, U.K.
4. Faculty of Sport and Exercise Science, UCLan University of Cyprus, Pyla 7080, Cyprus
5. Montenergin Sports Academy, Podgorica, Montenegro
6. Department of Physical Education and Sport, Faculty of Sport Sciences, Burdur Mehmet Akif Ersoy University, Burdur 15030, Turkey
7. M2S (Laboratoire Mouvement, Sport, Santé), Université Rennes, Rennes, France
8. Institut International des Sciences du Sport (2I2S), 35850, Irodouer, France
9. Department of Sport and Sport Science, Exercise and Human Movement Science, University of Freiburg, 79102 Freiburg, Germany

**Correspondence**

Name: Prof. Urs Granacher

Address: Department of Sport and Sport Science, Exercise and Human Movement Science, University of Freiburg, 79102 Freiburg, Germany

Email: [Urs.Granacher@sport.uni-freiburg.de](mailto:Urs.Granacher@sport.uni-freiburg.de)

<https://orcid.org/0000-0002-7095-813X>

**Sports Medicine**

**Supplementary Material 1**

Additional data from the included studies data (n=17)

| **Studies** | **Outcome measures** | **Tests** | **FIFA 11+** | | **n** | **Control** | | **n** |
| --- | --- | --- | --- | --- | --- | --- | --- | --- |
|  |  |  | **Pre ± SD** | **Post ± SD** |  | **Pre ± SD** | **Post ± SD** |  |
| Akbari et al. [1] | Vertical jump | VJ (cm) | 45.33 ± 5.06 | 51 ± 4.95 | 12 | 44.25 ± 4.78 | 44.58 ± 3.75 | 12 |
| Arede et al. [2] | Dynamic balance | YBT-R | 92.42± 9.02 | 99.55± 9.33 | 15 | 91.94± 7.58 | 97.53± 4.17 | 15 |
|  | Dynamic balance | YBT-L | 95.40 ± 8.08 | 101.31 ± 8.66 | 15 | 92.96 ± 8.02 | 97.69 ± 5.54 | 15 |
|  | COD | T-TEST (s) | 12.72 ± 1.41 | 12.52 ± 2.22 | 15 | 12.00 ± 0.67 | 12.13 ± 0.95 | 15 |
|  | Speed | 20m (s) | 3.56 ± 0.41 | 3.32 ± 0.46 | 15 | 3.44 ± 0.22 | 3.42 ± 0.2 | 15 |
|  | Vertical jump | CMJ (cm) | 27.89 ± 8.01 | 27.17 ± 7.37 | 15 | 23.85 ± 3.69 | 22.57 ± 5.08 | 15 |
|  | Vertical jump | VJ (cm) | 33.73 ± 8.07 | 32.20 ± 8.30 | 15 | 26.27 ± 8.05 | 26.53 ± 4.53 | 15 |
| Asgari et al. [3] | COD | IAT (s) | 17.27 ± 0.55 | 16.7 ± 0.77 | 29 | 17.27 ± 0.58 | 16.92 ± 0.54 | 30 |
| Ayala et al. [4] | Dynamic balance | YBT-A (% of L L) | 67.5 ± 6.1 | 67.3 ± 6.3 | 10 | 70.5 ± 5.3 | 64.8 ± 5.2 | 11 |
|  | Dynamic balance | YBT-PM (% of L L) | 112.9 ± 4.8 | 115.3 ±2.8 | 10 | 112 ± 7.8 | 109.2 ± 6.3 | 11 |
|  | Dynamic balance | YBT-PL (% of L L) | 109.2 ± 5.3 | 109.5 ± 4.9 | 10 | 108.3 ± 8.5 | 106.5 ± 5.7 | 11 |
|  | COD | IAT (s) | 16.66 ± 1.16 | 16.26 ± 0.83 | 10 | 16.57 ± 0.98 | 16.4 ± 1.37 | 11 |
|  | Speed | 10m (s) | 1.93 ± 0.13 | 2.3 ± 0.23 | 10 | 1.82 ± 0.28 | 2.9 ± 0.33 | 11 |
|  | Speed | 20m (s) | 3.3 ± 0.18 | 3.24 ± 0.15 | 10 | 3.28 ± 0.15 | 3.31 ± 0.23 | 11 |
|  | Vertical jump | DJ (cm) | 26.7 ± 2.2 | 26.6 ± 3.5 | 10 | 24.1 ± 2.2 | 22.4 ± 5.5 | 11 |
| Costa Silva et al. [5] | Vertical jump | CMJ (cm) | 45.84 ± 3.91 | 51.04 ± 2.88 | 10 | 43.56 ± 4.41 | 44.66 ± 3.91 | 10 |
|  | Vertical jump | SJ (cm) | 41.33 ± 2.7 | 46.67 ± 2.71 | 10 | 40.15 ± 3.61 | 40.66 ± 3.57 | 10 |
| Daneshjoo et al. [6] | Dynamic balance | SEBT (% of L L) | 97.2 ± 9.4 | 103.9 ± 5.6 | 12 | 97.7 ± 5.9 | 98.4 ± 3.9 | 12 |
| Daneshjoo et al. [7] | COD | IAT (s) | 16.2 ± 0.3 | 14.4 ± 0.3 | 12 | 16.5 ± 1.2 | 16.9 ± 1.2 | 12 |
|  | Speed | 10m (s) | 5.6 ± 0.4 | 5.3 ± 0.4 | 12 | 5.7 ± 0.6 | 6.1 ± 0.5 | 12 |
|  | Speed | 20m (s) | 3.1 ± 0.4 | 2.7 ± 0.3 | 12 | 3.1 ± 0.1 | 3.2 ± 0.1 | 12 |
|  | Vertical jump | VJ (cm) | 47 ± 6.3 | 50.7 ± 7.9 | 12 | 46.6 ± 5.4 | 48.9 ± 7.6 | 12 |
| Foqha et al. [8] | COD | MAT (s) | 6.43 ± 0.13 | 5.51 ± 0.08 | 13 | 6.57 ± 0.07 | 6.22 ± 0.13 | 12 |
|  | Speed | 10m (s) | 1.95 ± 0.18 | 1.88 ± 0.07 | 13 | 1.81 ± 0.03 | 1.87 ± 0.02 | 12 |
|  | Speed | 20m (s) | 1.81 ± 0.03 | 3.24 ± 0.03 | 13 | 3.13 ± 0.03 | 3.18 ± 0.03 | 12 |
| Hwang et al. [9] | COD | AAT-R (s) | 9.0 ± 0.2 | 8.8 ± 0.3 | 10 | 9.0 ± 0.2 | 9 ± 0.2 | 10 |
|  | COD | AAT-L (s) | 9.0 ± 0.2 | 8.6 ± 0.3 | 10 | 8.9 ± 0.4 | 9 ± 0.3 | 10 |
|  | Speed | 10m (s) | 1.7 ± 0.0 | 1.7 ± 0.01 | 10 | 1.7 ± 0.0 | 1.7 ± 0.01 | 10 |
|  | Speed | 30m (s) | 4.2 ± 0.1 | 4.1 ± 0.01 | 10 | 4.2 ± 0.1 | 4.2 ± 0.1 | 10 |

| **Supplementary Material 1.** Continued. | | | | | | | | |
| --- | --- | --- | --- | --- | --- | --- | --- | --- |
| Impellizzeri et al. [10] | Dynamic balance | SEBT (% of L L) | 90 ± 5.9 | 91.7 ± 6.1 | 42 | 89.9 ± 6.2 | 90.6 ± 6.5 | 39 |
|  | COD | T-test (s) | 10.7 ± 0.3 | 10.4 ± 0.5 | 42 | 10.6 ± 0.4 | 10.5 ± 0.5 | 39 |
|  | Speed | 20m (s) | 3.31 ± 0.12 | 3.22 ± 0.12 | 42 | 3.29 ± 0.14 | 3.24 ± 0.12 | 39 |
|  | Vertical jump | VJ (cm) | 46.7 ± 6.7 | 47.4 ± 7 | 42 | 45.3 ± 6.9 | 45.2 ± 7.5 | 39 |
| Nawed et al. [11] | COD | T-test (s) | 12.10 ± 1.15 | 11.64 ± 1.6 | 29 | 13.07 ± 0.89 | 13.37 ± 0.89 | 28 |
|  | COD | IAT (s) | 17.78 ± 0.51 | 17.19 ± 1.16 | 29 | 16.99 ± 2.26 | 16.73 ± 2.42 | 28 |
|  | Speed | 20m (s) | 3.47 ± 0.16 | 3.09 ± 0.14 | 29 | 3.48 ± 0.10 | 3.5 ± 0.14 | 28 |
|  | Vertical jump | VJ (cm) | 37.44 ± 14.86 | 42.11 ± 14.67 | 29 | 37.70 ± 6.42 | 37.3 ± 6.11 | 28 |
| Pardos-Mainer et al. [12] | Dynamic balance | YBT-A (% of L L) | 95.3 ± 4.65 | 94.6± 5.12 | 19 | 96.1 ± 3.03 | 94.6± 3.29 | 17 |
|  | Dynamic balance | YBT-PM (% of L L) | 92.3 ± 7.48 | 93.6 ± 3.5 | 19 | 89.8 ± 8.44 | 90.3 ± 8.37 | 17 |
|  | Dynamic balance | YBT-PL (% of L L) | 93.1 ± 5.53 | 95.4 ± 4.34 | 19 | 90.4 ± 6.13 | 92.2 ± 6.39 | 17 |
|  | COD | V-CUT (s) | 7.89 ± 0.33 | 8.09 ± 0.33 | 19 | 7.85 ± 0.39 | 8.16 ± 0.31 | 17 |
|  | Vertical jump | DJ (cm) | 21.2 ± 3.44 | 23.3 ± 3.5 | 19 | 23.3 ± 4.86 | 24.7 ± 4.55 | 17 |
|  | Vertical jump | CMJ (cm) | 20 ± 3.68 | 22.1 ± 3.39 | 19 | 22.8 ± 3.12 | 23.1 ± 6.28 | 17 |
| Parsons et al. [13] | Dynamic balance | YBT-R (% of L L) | 94.8 ± 7 | 94.2 ± 6.8 | 25 | 97.2 ± 7.5 | 93.4 ± 8.1 | 25 |
|  | Dynamic balance | YBT-L (% of L L) | 96.4 ± 6.4 | 94.5 ± 6.7 | 25 | 97.2 ± 6.9 | 94.6 ± 7.1 | 25 |
|  | COD | T-test (s) | 14.2 ± 1.1 | 13.9 ± 0.8 | 25 | 14.2 ± 1.2 | 13.8 ± 1.2 | 25 |
|  | Vertical jump | VJ (cm) | 36.9 ± 5.2 | 37.2 ± 4.1 | 25 | 36.4 ± 5.1 | 36.1 ± 6.1 | 25 |
| Robles-Palazón et al. [14] | Dynamic balance | YBT-A (% of L L) | 68.1 ± 6.9 | 67.8 ± 6.2 | 10 | 69.7 ± 4.9 | 65.8 ± 5.2 | 11 |
|  | Dynamic balance | YBT-PM (% of L L) | 113.7 ± 5.3 | 115.3 ± 2.8 | 10 | 110.4 ± 6.1 | 109.3 ± 6.4 | 11 |
|  | Dynamic balance | YBT-PL (% of L L) | 109.9 ± 4.6 | 109.5 ± 4.9 | 10 | 106.5 ± 5.7 | 106.6 ± 5.7 | 11 |
|  | Speed | 10m (s) | 1.89 ± 0.1 | 2.03 ± 0.23 | 10 | 1.81 ± 0.29 | 1.99 ± 0.33 | 11 |
|  | Speed | 20m (s) | 3.27 ± 0.15 | 3.24 ± 0.15 | 10 | 3.27 ± 0.16 | 3.31 ± 0.23 | 11 |
|  | Vertical jump | VJ (cm) | 26.9 ± 3.5 | 26.6 ± 3.5 | 10 | 24.3 ± 2.4 | 22.1 ± 5.5 | 11 |
| Sharifatpour et al. [15] | Vertical jump | BOSCO I | 89.97 ± 3.63 | 93.28 ± 2.79 | 20 | 88.93 ± 3.82 | 89.86 ± 2.85 | 20 |
| Zarei et al. [16] | COD | IAT (s) | 19.1 ± 0.9 | 18.3 ± 0.8 | 34 | 18.4 ± 1.0 | 18.1 ± 0.7 | 32 |
|  | Speed | 9.6m (s) | 1.8 ± 0.1 | 1.8 ± 0.2 | 34 | 1.7 ± 0.1 | 1.7 ± 0.1 | 32 |
|  | Vertical jump | VJ (cm) | 41.1 ± 7.5 | 45.0 ± 6.9 | 34 | 46.7 ± 4.8 | 47.8 ± 4.7 | 32 |
| Zhou et al. [17] | COD | SR (s) | 36.01 ± 2.52 | 33.51 ± 1.55 | 10 | 36.69 ± 2.16 | 35.25 ± 2.55 | 10 |
|  | Speed | 30m (s) | 4.81 ± 0.26 | 4.39 ± 0.26 | 10 | 5.11 ± 0.20 | 4.6 ± 0.17 | 10 |

*YBT-R= Y Balance Test- Right, YBT-L= Y Balance Test- Left, VJ= Vertical jump, MAT= Modified agility test, IAT= Illinois agility test, SR= Shuttle run test, SEBT= Star Excursion Balance Test,* *VDJ= Vertical drop jump, CMJ= Countermovement jump, AAT=* *Arrow agility test, SJ= squat jump, COD= Change of direction, % of L L= percentage of leg length, n=number of subjects, SD= Standard deviation, s= Seconds,* A= Anterior, PM=Posteromedial, PL= Posterolateral, cm= Centimetres*.*

**References**

[1] Akbari H, Sahebozamani M, Daneshjoo A, Amiri-Khorasani M. Effect of the FIFA 11+ programme on vertical jump performance in elite male youth soccer players. Montenegrin J. Sport. Sci. Med.2018;7(2):17–22. https://doi.org/10.26773/mjssm.180903.

[2] Arede J, Shang X, Moran J, Calleja-González J, Travassos B, Madruga-Parera M, Leite N. Does the FIFA 11 + warm-up program positively shape both the physical and small-sided game performance of youth football players?. Intell. Sport. Heal.2025;1(1):22–29. https://doi.org/10.1016/j.ish.2024.12.004.

[3] Asgari M, Alizadeh M H, Shahrbanian S, Nolte K, Jaitner T. Effects of the FIFA 11+ and a modified warmup programme on injury prevention and performance improvement among youth male football players. PLoS One2022;17(10 October):1–11. https://doi.org/10.1371/journal.pone.0275545.

[4] Ayala F, Pomares-Noguera C, Robles-Palazón F J, Del Pilar García-Vaquero M, Ruiz-Pérez I, Hernández-Sánchez S, De Ste Croix M. Training Effects of the FIFA 11+ and Harmoknee on Several Neuromuscular Parameters of Physical Performance Measures. Int. J. Sports Med.2017;38(4):278–89. https://doi.org/10.1055/s-0042-121260.

[5] Costa Silva J R L, Silva J F, Salvador P C do N, Freitas C D la R. O efeito do “FIFA 11+” na performance de saltos verticais em atletas de futebol. Rev. Bras. Cineantropometria e Desempenho Hum.2015;17(6):733. https://doi.org/10.5007/1980-0037.2015v17n6p733.

[6] Daneshjoo A, Mokhtar A H, Rahnama N, Yusof A. The Effects of Comprehensive Warm-Up Programs on Proprioception, Static and Dynamic Balance on Male Soccer Players. PLoS One2012;7(12):e51568. https://doi.org/10.1371/journal.pone.0051568.

[7] Daneshjoo A, Mokhtar A H, Rahnama N, Yusof A. Effects of the 11+ and Harmoknee warm-up programs on physical performance measures in professional soccer players. J. Sport. Sci. Med.2013;12(3):489–96.

[8] Foqha B M, Schwesig R, Ltifi M A, Bartels T, Hermassi S, Aouadi R. A 10-week FIFA 11+ program improves the short-sprint and modified agility T-test performance in elite seven-a-side soccer players. Front. Physiol.2023;14(November):1–11. https://doi.org/10.3389/fphys.2023.1236223.

[9] Hwang J, Kim J. Effect of fifa 11+ training program on soccer-specific physical performance and functional movement in collegiate male soccer players: A randomized controlled trial. Exerc. Sci.2019;28(2):141–49. https://doi.org/10.15857/ksep.2019.28.2.141.

[10] Impellizzeri F M, Bizzini M, Dvorak J, Pellegrini B, Schena F, Junge A. Physiological and performance responses to the FIFA 11+ (part 2): A randomised controlled trial on the training effects. J. Sports Sci.2013;31(13):1491–1502. https://doi.org/10.1080/02640414.2013.802926.

[11] Nawed A, Khan I A, Jalwan J, Nuhmani S, Muaidi Q I. Efficacy of FIFA 11 + training program on functional performance in amateur male soccer players. J. Back Musculoskelet. Rehabil.2018;31(5):867–70. https://doi.org/10.3233/BMR-171034.

[12] Pardos-Mainer E, Casajús J A, Gonzalo-Skok O. Adolescent female soccer players’ soccer-specific warm-up effects on performance and inter-limb asymmetries. Biol. Sport2019;36(3):199–207. https://doi.org/10.5114/biolsport.2019.85453.

[13] Parsons J L, Carswell J, Nwoba I M, Stenberg H. Athlete Perceptions and Physical Performance Effects of the Fifa 11+ Program in 9-11 Year-Old Female Soccer Players: a Cluster Randomized Trial. Int. J. Sports Phys. Ther.2019;14(5):740–52. https://doi.org/10.26603/ijspt20190740.

[14] Robles-Palazón F J, Pomares-Noguera C, Ayala F, Hernández-Sánchez S, Martínez-Romero M T, De Baranda S P, Wesolek I. Acute and Chronic Effects of the Fifa 11+ on Several Physical Performance Measures in Adolescent Football Players. Eur. J. Hum. Mov.2016;36:116–36.

[15] Sharifatpour R, Abbasi H, Alizadeh M H, Abedinzadeh S, Machado S. Effect of 12 weeks of modified FIFA 11+ training on functional factors of male beach soccer players: A Randomized Controlled Trial study. Cuad. Psicol. del Deport.2024;24(3):265–80. https://doi.org/10.6018/cpd.584601.

[16] Zarei M, Abbasi H, Daneshjoo A, Barghi T S, Rommers N, Faude O, Rössler R. Long-term effects of the 11+ warm-up injury prevention programme on physical performance in adolescent male football players: a cluster-randomised controlled trial. J. Sports Sci.2018;36(21):2447–54. https://doi.org/10.1080/02640414.2018.1462001.

[17] Zhou X, Luo A, Wang Y, Zhang Q, Zha Y, Wang S, Ashton C, Andamasaris J E, Wang H, Wang Q. The Effect of FIFA 11+ on the Isometric Strength and Running Ability of Young Soccer Players. Int. J. Environ. Res. Public Health2022;19(20):13186. https://doi.org/10.3390/ijerph192013186.
