# Supplementary material for: Effects of the FIFA 11 + Program on Physical Fitness in Youth and Adult Soccer Players: A Systematic Review and Meta-analysis
Source: Sports Med. 2025 Nov 25;56(2):521–41. doi: 10.1007/s40279-025-02346-8 (PMC12982247; doi:10.1007/s40279-025-02346-8)
Supplement: Supplementary file 2 — Supplementary file2 (DOCX 206 kb) [file 40279_2025_2346_MOESM2_ESM.docx]

**Supplementary Material**

**Effects of the FIFA 11+ Program on Physical Fitness in Youth and Adult Soccer Players: A Systematic Review and Meta-Analysis**

**Short title:** FIFA 11+ and Physical Fitness in Soccer Players: A Meta-Analysis

Ibnu Noufal Kambitta Valappil^1^; Karuppasamy Govindasamy^2^; Gavoutamane Vasanthi^1^; Masilamani Elayaraja^1^; Cain C.T.Clark^3^; Koulla Parpa^4^; Borko Katanic^5^; Hüseyin Şahin Uysal^6^; [Hassane Zouhal](https://link.springer.com/article/10.1186/s40798-025-00831-y#auth-Hassane-Zouhal-Aff8-Aff9)^7,8^; Urs Granacher^9^**^*^**

1. Department of Physical Education and Sports, Pondicherry University, 605014 Puducherry, India
2. Department of Sports, Recreation and Wellness, Symbiosis International (Deemed University), Hyderabad Campus, Modallaguda (V), Nandigama (M), Rangareddy, 509217 Telangana, India
3. College of Life Sciences, Birmingham City University, B15 3TN, U.K.
4. Faculty of Sport and Exercise Science, UCLan University of Cyprus, Pyla 7080, Cyprus
5. Montenergin Sports Academy, Podgorica, Montenegro
6. Department of Physical Education and Sport, Faculty of Sport Sciences, Burdur Mehmet Akif Ersoy University, Burdur 15030, Turkey
7. M2S (Laboratoire Mouvement, Sport, Santé), Université Rennes, Rennes, France
8. Institut International des Sciences du Sport (2I2S), 35850, Irodouer, France
9. Department of Sport and Sport Science, Exercise and Human Movement Science, University of Freiburg, 79102 Freiburg, Germany

**Correspondence**

Name: Prof. Urs Granacher

Address: Department of Sport and Sport Science, Exercise and Human Movement Science, University of Freiburg, 79102 Freiburg, Germany

Email: [Urs.Granacher@sport.uni-freiburg.de](mailto:Urs.Granacher@sport.uni-freiburg.de)

<https://orcid.org/0000-0002-7095-813X>

**Sports Medicine**

**Supplementary Material 2**


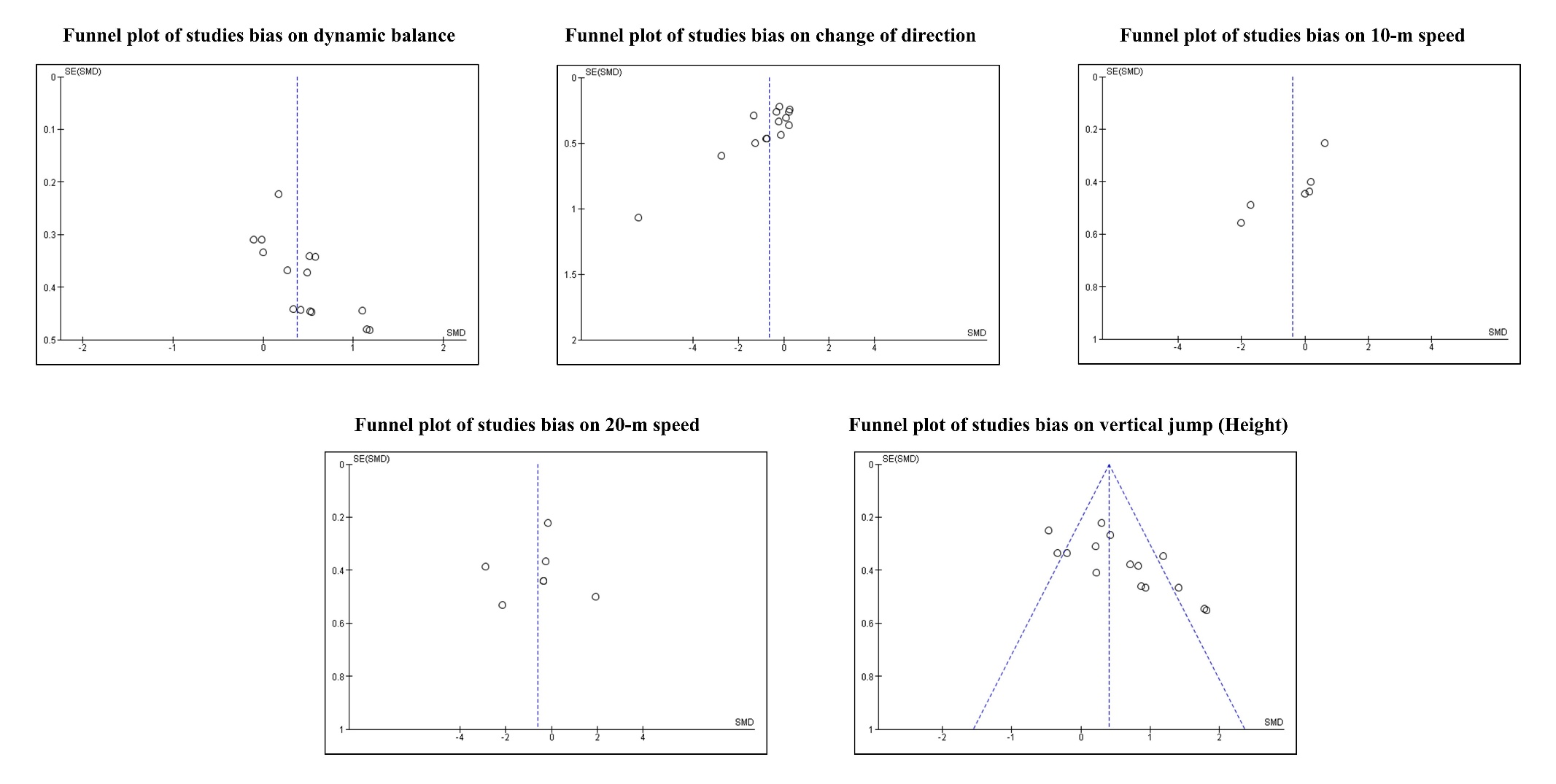


**Supplementary figure 1.** Funnel plots were generated to assess potential publication bias for dynamic balance, change-of-direction speed, linear sprints (10-m and 20-m), and vertical jump height.
